# Evolution of Skull and Mandible Shape in Cats (Carnivora: Felidae)

**DOI:** 10.1371/journal.pone.0002807

**Published:** 2008-07-30

**Authors:** Per Christiansen

**Affiliations:** Department of Vertibrates, Zoological Museum, Copenhagen, Denmark; Smithsonian Institution, United States of America

## Abstract

The felid family consists of two major subgroups, the sabretoothed and the feline cats, to which all extant species belong, and are the most anatomically derived of all carnivores for predation on large prey with a precision killing bite. There has been much controversy and uncertainty about why the skulls and mandibles of sabretoothed and feline cats evolved to become so anatomically divergent, but previous models have focused on single characters and no unifying hypothesis of evolutionary shape changes has been formulated. Here I show that the shape of the skull and mandible in derived sabrecats occupy entirely different positions within overall morphospace from feline cats, and that the evolution of skull and mandible shape has followed very different paths in the two subgroups. When normalised for body-size differences, evolution of bite forces differ markedly in the two groups, and are much lower in derived sabrecats, and they show a significant relationship with size and cranial shape, whereas no such relationship is present in feline cats. Evolution of skull and mandible shape in modern cats has been governed by the need for uniform powerful biting irrespective of body size, whereas in sabrecats, shape evolution was governed by selective pressures for efficient predation with hypertrophied upper canines at high gape angles, and bite forces were secondary and became progressively weaker during sabrecat evolution. The current study emphasises combinations of new techniques for morphological shape analysis and biomechanical studies to formulate evolutionary hypotheses for difficult groups.

## Introduction

The Felidae is made up of two distinct evolutionary lineages, the modern cats, often referred to as the Felinae or true cats, and the extinct sabretoothed cats in the subfamily Machairodontinae [1], [2]. The cat lineage is highly anatomically derived for predation, but the great anatomical divergence within the group indicates that evolutionary selection has been very different. Modern cats are characterized by being anatomically derived for predation with a powerful precision killing bite 3–5. Sabretoothed cats were often highly different from modern cats in cranio-mandibular morphology [1], [2], [6], and it has been a subject of much controversy and uncertainty about why the skulls and mandibles of sabretoothed and feline cats evolved to become so anatomically divergent [6]–[9], but today, it is widely held that sabrecats probably used their large canines in a shearing bite to the throat of prey, severing nerves and blood vessels, causing rapid, if not instant collapse [2], [10], [11]. Although the particulars of the predatory sequence is unknown among sabrecats, this killing style probably also required a precision bite [2], [6], [11]–[13].

Analyses have traditionally focused on singular characters to understand sabretooth morphology, such as the morphology of the mastoid and paroccipital region [11], [12], or adaptations for attaining a large gape, such as a ventrally deflected glenoid fossa and reduced coronoid process [6], [10], [14]. However, there is still no comprehensive theory of the selective forces which governed the changes in shape of the skull and mandible as integrated units during the course of felid evolution, and how this affected the function and performance of these predators. Although portions of the skull may evolve independently [15], [16], there is ample evidence that the skull is optimized to function as a coherent mechanical unit [17]–[21]. In this paper, I demonstrate and illustrate the evolutionary shape changes in the skull and mandible across the entire felid family, by using new approaches that model shape changes in the entire skull and mandible simultaneously, and also address how this affected the mechanical performance during the killing bite, by comparing estimated bite forces among the species using a new technique, that allows comparison of bite forces irrespective of differences in body size. This combined approach sheds new light onto the evolutionary history of the unusual felid predators, and allows formulation of a more comprehensive theory of how and why the derived members of each subgroup of cats eventually became so morphologically different. It also shows that large changes in selective driving forces are possible within a relatively narrow group of mammals, in this case a family of carnivorans.

## Results

**Figure 1:**
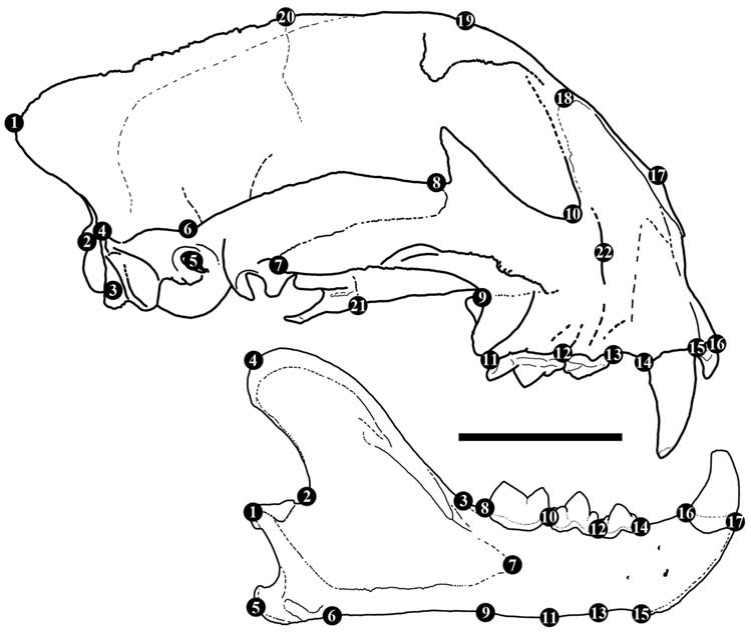
The 22 (cranial) and 17 (mandibular) morphologically homologous landmarks used in the analysis of felid craniomandibular shape. Skull and mandible of a puma (*Puma concolor*; ♂; CN3435) illustrating the various landmarks. Landmarks on skull are: apex of supraoccipital (1); dorsoventral extent of occipital condyle (2, 3); transition of horizontal temporal bridge and occiput (4); centre of acoustic meatus (5); posterior extent of zygomatic arch (squamous portion) (6); ventral (7) and dorsal (8) squamous and jugal suture of zygomatic arch; ventral sutural connection of jugal to maxilla (9); ventro-arboreal extent of orbital foramen (10); anteroposterior extent of P^4^ (11–12), P^3^ (12–13), and C^1^ (14–15) along gumline; arboreal extent of premaxilla at alveolar margin of I^3^ (16); apex of nasal (17); dorsal nasal-maxilla suture (18); apex of skull at postorbital frontal process (19); apex of skull at coronal suture (20); ventral palatine-pterygoid suture (21); centre of infraorbital foramen (22). Landmarks on mandible are: centre of mandibular condyle (1); anteroposterior extent of basal portion of coronoid process (2–3); apex of coronoid process (4); anteroposterior extent (5–6) and ventral deflection of angular process; anterior extent of mandibular fossa (7); length of M_1_ (8–10), P_4_ (10–12), and P_3_ (12–14); dorsoventral depth of horizontal ramus posterior to M_1_ (8–9), P_4_ (10–11), and posterior (12–13) and anterior (14–15) to P_3_; anteroposterior diameter of C_1_ (16–17). Scale bar equals 5 cm.

**Figure 2:**
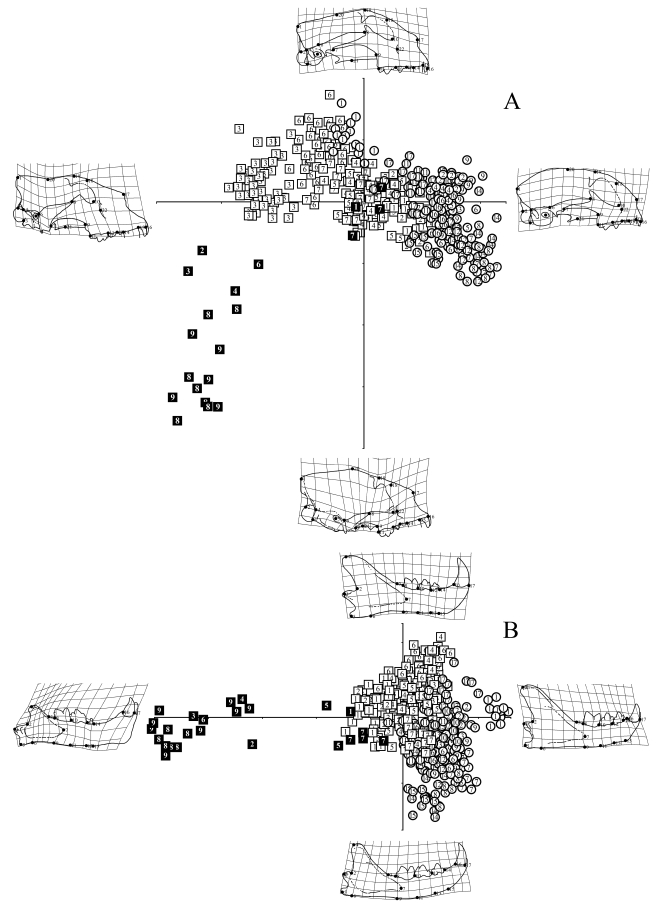
Skull and mandible shapes in cats as illustrated by 22 (cranium) and 17 (mandible) landmarks. (A) Scatter plots of relative warps 1 and 2 for shape changes in the skulls of felids, along with morphological standards at the axis apices. Relative warps 1 and 2 summarize 40.1% and 20.0%, respectively, of sample variation in the analysis. (B) Scatter plots of relative warps 1 and 2 for shape changes in the mandibles of felids, , along with morphological standards at the axis apices. Relative warps 1 and 2 summarize 50.7% and 18.2%, respectively, of sample variation in the analysis. Symbols: Open circles, non-pantherine (“small”) felids: 1, *Acinonyx jubatus*; 2, *Caracal caracal*; 3, *Catopuma temmincki*; 4, *Felis chaus*; 5, *Felis silvestris*; 6, *Leopardus pardalis*; 7, *Leopardus tigrina*; 8, *Leopardus wiedii*; 9, *Leptailurus serval*; 10, *Lynx canadensis*; 11, *Lynx lynx*; 12, *Oncifelis geoffroyi*; 13, *Pardofelis marmorata*; 14, *Prionailurus bengalensis*; 15, *Prionailurus planiceps*; 16, *Prionailurus viverrinus*; 17, *Puma concolor*. Open squares, pantherine felids: 1, *Neofelis diardi*; 2, *Neofelis nebulosa*; 3, *Panthera leo*; 4, *Panthera onca*; 5, *Panthera pardus*; 6, *Panthera tigris*; 7, *Panthera uncia*. Closed squares, sabertoothed felids: 1, *Dinofelis barlowi*; 2, *Epimachairodus giganteus*; 3, *Homotherium crenatidens*; 4, *Homotherium serum*; 5, *Machairodus aphanistus* (mandible only); 6, *Megantereon cultridens*; 7, *Paramachairodus ogygia*; 8, *Smilodon fatalis*; 9, *Smilodon populator*.

Based on warp analysis of 22 cranial and 17 mandibular landmarks (Fig. 1), it is evident that the entire shape of the skull and mandible in derived sabrecats became dramatically different from those of extant cats during the course of evolution, and they collectively occupy an entirely separate portion of overall morphospace from any extant felid (Fig. 2A,B). Derived sabrecats primarily group distinctly from all extant cats on relative warp 2, and differences within derived sabrecats appear related to the length of the upper canines, since dirk-toothed *Smilodon* sp. group separately with lower relative warp scores from other derived sabrecats, such as scimitar-toothed *Homotherium* sp. and *Epimachairodus*. Relative warp 2 is primarily related to dorsoventral skull shape, and specimens with lower warp scores have a dorsoventrally much taller and anteroposteriorly more compact skull, ventrally deflected glenoid fossa, greatly curved and anteroventrally compressed and dorsoventrally tall zygomatic arch, elevated facial portion of the skull, and abbreviated mid-section of the skull. They also have enlarged external nares and distinct posterior retraction of the infraorbital foramen, posteroventral deflection of the ventral orbital rim, and slightly smaller and dorsally deflected occipital condyles. In contrast, primitive sabrecats such as puma-sized *Paramachairodus* and jaguar-sized *Dinofelis* group with the extant clouded leopard and Diard's clouded leopard (genus *Neofelis*), a taxon which, uniquely among extant felids, is known to have numerous characters in common with primitive sabrecats [14], [22], [23]. This demonstrates that it is not in accord with evolutionary morphology to divide the cats into two groups which are inferred to differ markedly in cranio-mandibular morphology, because some members of the feline group (*Neofelis*) and primitive members of the sabretooth group converge morphologically. The enormous divergence of later sabretooths was a result of distinct evolutionary selective forces operating within the group, and not an inherent characteristic of the group.

In contrast to derived sabrecats, modern cats differ primarily in skull shape along relative warp 1, with very large species (lion, tiger) having low warp scores, implying an elongate snout region, anteroposteriorly compressed mid-part of the skull, elongate and straighter posterior part of the skull, more dorsoventrally elongate orbital aperture, more powerfully built zygomatic arch, and slightly lowered glenoid fossa. It has previously been suggested that skull morphology in modern cats will divide these into two groups; large species, encompassing the *Panthera* cats (lion, jaguar, leopard, tiger, and snow leopard), and small cats, respectively, with some taxa (puma, *Neofelis*) occupying intermediate positions between the two [24]; this would imply different evolutionary selective forces for skull shape in small and large cats. However, traditional approaches do not actually study shape [25], and the current analysis of skull shape does not support such a dichotomy. Rather, the shape of the skull along relative warp 1 represents a continuum which covaries highly significantly with overall skull size, here defined as condylobasal skull length, whereas relative warp 2 does not (Table 1). There is no discernible division between large and small cats, and among *Panthera*, the smaller species (leopard, jaguar, snow leopard) have higher relative warp 1 scores than the lion and tiger, and among so-called small cats, larger species (e.g., *Lynx* sp. fishing cat; puma) have higher warp scores than smaller species (e.g., Geoffroy's cat; leopard cat; margay; see also Supplementary Information). Thus, the utility of felid skull shape characters in systematic analyses [26] is questionable, owing to shape being highly size-dependent and not readily quantifiable into discrete systematic characters. Among sabrecats, the shape of the skull along relative warp 1 is also size-dependent, and there appears to be a size-dependence along relative warp 2 as well (Table 1), albeit non-significantly so, primarily owing to the differences between scimitar-toothed and dirk-toothed forms. Thus, among modern cats, the uniformity of skull shape when correcting for size indicates similar evolutionary selective forces; as will be shown below, these were most likely mechanical reasons.

**Table 1:** Interspecific Reduced Major Axis regression lines for analyses of skull size (log_10_ condylobasal length in mm [CBL]), relative upper canine height to CBL (C/CBL; arcsine transformed ratio), Bite Force Quotients at the canine (BFQ; in Newtons), and associated skull shape (Relative warps 1 and 2, [Relw1], and [Relw2], respectively); mandible size (log_10_ mandible length in mm [MAN]), and associated mandible shape (Relative warps 1 and 2, [Relw1], and [Relw2], respectively) along with correlation coefficients (r), standard errors of the estimate (SEE), and significance of the regression.

Sample	n	X	Y	α±95% CI	β±95% CI	r	SEE	F	p
Extant cats	24	CBL	Relw1	0.635±0.112	−0.283±0.053	0.907	0.020	101.604	<0.001
Sabrecats	8	CBL	Relw1	1.352±0.632	−0.591±0.260	0.898	0.026	24.986	0.002
Extant cats	24	CBL	Relw2	0.383±0.166	−0.178±0.078	0.089	0.030	0.177	0.678*ns*
Sabrecats	8	CBL	Relw2	1.311±1.064	−0.564±0.428	0.631	0.044	3.970	0.093*ns*
Extant cats	24	C/CBL	Relw1	0.533±0.182	−0.021±0.007	0.564	0.039	10.282	0.004
Sabrecats	8	C/CBL	Relw1	0.345±0.252	−0.009±0.007	0.650	0.052	4.396	0.081*ns*
Extant cats	24	C/CBL	Relw2	0.318±0.134	−0.013±0.006	0.222	0.029	1.142	0.297*ns*
Sabrecats	8	C/CBL	Relw2	0.192±0.260	−0.005±0.008	0.201	0.054	0.253	0.633*ns*
Extant cats	24	CBL	BFQ	−37.759±62.149	66.492±29.398	0.012	11.077	0.003	0.954*ns*
Sabrecats	8	CBL	BFQ	780.366±178.770	298.312±435.124	0.800	18.087	10.672	0.017
Extant cats	24	BFQ	Relw1	111.422±6.153	−234.802±103.813	0.010	11.077	0.002	0.964*ns*
Sabrecats	8	BFQ	Relw1	97.769±34.234	504.987±343.544	0.732	20.532	6.937	0.039
Extant cats	24	BFQ	Relw2	105.227±4.851	−373.795±165.266	0.010	11.078	0.002	0.962*ns*
Sabrecats	8	BFQ	Relw2	87.435±37.197	528.767±470.172	0.456	26.836	1.573	0.256*ns*
Extant cats	24	MAN	Relw1	0.335±0.129	−0.159±0.066	0.355	0.025	3.165	0.089*ns*
Sabrecats	9	MAN	Relw1	1.464±1.157	−0.699±0.505	0.588	0.059	3.694	0.096*ns*
Extant cats	24	MAN	Relw2	−0.424±0.097	0.208±0.050	0.843	0.018	54.196	<0.001
Sabrecats	9	MAN	Relw2	−0.384±0.274	0.165±0.147	0.063	0.017	0.028	0.871*ns*

It has been suggested that one difference between sabretoothed cats and modern cats is that in sabrecats, skull shape is primarily related to skull size, whereas shape is more closely related to upper canine size among sabrecats [27]. This is corroborated to some extent by this study, but most derived sabrecats are larger than primitive ones, clouding this image. Additionally, among extant cats, the upper canine also becomes longer relative to condylobasal skull length with increasing skull size (β = 0.481±0.164; r = 0.689; F = 19.929; p<0.001), but outliers are prevalent, in particular *Neofelis* sp., which have proportionally very long upper canines, and the lion, which has short upper canines. This implies curvilinearity of the sample, as indicated by significantly (p<0.05) higher correlation (r = 0.752) with application of polynomial regression. Among sabretoothed felids, the ratio of upper canine length to condylobasal skull length also increases with increasing skull size (β = 1.504±1.251; r = 0.552), but owing to small sample size (n = 8) and great heterogeneity of proportional canine size between the dirk-toothed (*Megantereon*, *Smilodon*) and scimitar-toothed (*Epimachairodus*, *Homotherium*) species, the regression equation is non-significant (F = 2.635; p = 0.156). No curvilinearity is present in this sample, as indicated by a non-significantly different correlation coefficient with application of polynomial regression (r = 0.612; 0.30<p<0.40). Among extant cats, skull shape along relative warp 1 is related to the relative size of the upper canine (Table 1), but this is most likely a side effect of large cats having proportionally longer canines, and not the relative size of the canine *per se*. Among sabrecats, there is a tendency towards very long-toothed forms having lower warp scores along relative warp 1, and warp scores show a weak but non-significant relationship with skull size. However, as sabrecats differ in skull shape primarily along relative warp 2, and as this warp shows no relationship with skull size, the effect of upper canine length on skull shape is not linear. Rather, skull shape is a reflection of how derived a taxon is with respects to attaining a high gape; some of the derived species (*Epimachairodus*, *Homotherium*) have shorter upper canines, whereas *Megantereon* has enormous upper canines, but in some respects appears less derived than *Homotherium*.

Mandible shape displays the reverse pattern of skull shape (Fig. 2B; Table 1). Here, the sabrecats differ primarily along relative warp 1, and again the derived species occupy an entirely different part of overall morphospace from any extant species. Extant cats differ primarily along relative warp 2. Among extant cats, *Neofelis* sp. groups somewhat separately from other species, and the primitive sabrecats (*Dinofelis*, *Machairodus*, *Paramachairodus*) group with them, indicating that their overall mandibular morphology is similar. Derived sabrecats primarily differ from extant cats and also from primitive sabrecats in having an anteroposteriorly compressed posterior part of the mandible, but a concomitant elongate anterior part of the mandible, distinctly posterior deflected mandibular condyle, greatly dorsoventrally shortened coronoid process, which is also anteroposteriorly compressed, and greatly expanded mandibular symphysis. Other changes include reduction in P_3_ size, and anterior deflection of the anterior-most extent of the mandibular fossa. Among modern cats, the smaller species tend to have a strongly curved horizontal mandibular ramus, whereas large species (*Acinonyx*, *Neofelis*, *Panthera*, *Puma*), have a more rectangular ramus with a straight or even concave ventral profile. There is no systematic difference in dental size, or height of the coronoid process among extant cats. Relative warp 1 is not size-dependent in modern cats, whereas relative warp 2 is strongly size-dependent (Table 1). In sabrecats, the pattern is reverse, with relative warp 2 being entirely uncoupled from mandible size, and relative 1 showing a tendency towards size-dependency, but the equation is not significant owing to lower sample size, and the fact that some large sabrecats (*Dinofelis*, *Machairodus*) have primitive mandibles, whereas others (e.g., *Homotherium*, *Smilodon*) are highly derived.

**Figure 3:**
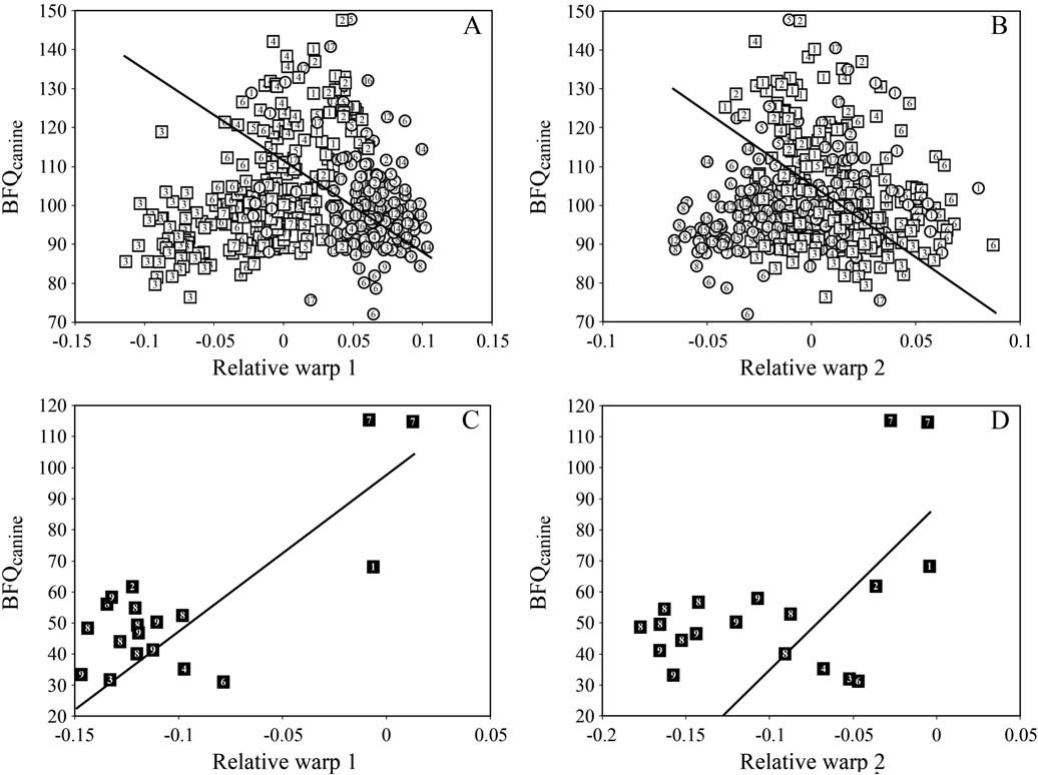
Bite force quotients against skull shape in felids. (A) Plots of bite force quotients at the canine (BFQ_canine_) against relative warps 1 and 2 in modern felids. Bite force quotients are entirely uncoupled from skull shape on both relative warps, and the regression lines are not even significant at the 90% level. (B) Plots of bite force quotients at the canine (BFQ_canine_) against relative warps 1 and 2 in extinct sabretoothed felids. Bite force quotients are significantly coupled to skull shape, although small sample size precludes assumptions of significance of the regression line at the 5% level for relative warp 2, but the regression is significant at the 10% level. Symbols as in Figure 1. Regression lines are interspecific Reduced Major Axis regression; for regression coefficients, see Table 1.

The maximal estimated bite forces at the canines normalised for differences in body size (the Bite Force Quotient or BFQ) is highly significantly higher among feline cats (F = 50.152; p<0.00001) than among sabrecats. The BFQ scores among modern cats are entirely uncoupled from skull size, whereas there is a significant correlation among sabrecats (Table 1). However, as noted above, this is a function of the highly modified skulls of derived sabrecats, not their size *per se* [see ref. 28]. Primitive sabrecats such as *Paramachairodus ogygia* and tiger-sized *Machairodus aphanistus* have much stronger bite forces that more derived forms (*Epimachairodus*), which again have stronger bite forces than the most derived forms, such as *Homotherium*, *Megantereon*, and *Smilodon*, which are of equal size to *Machairodus* [see supplementary information and ref. 28 for bite forces in *Machairodus aphanistus*]. This is also evident in that bite forces covary with skull shape among sabrecats (Table 1; Fig. 3C,D), although relative warp 2 is non-significant. This is probably a function of low sample size and the fact that *Machairodus aphanistus* could not be included in the skull shape analysis. Among extant cats, bite forces normalised for body size show absolutely no relationship with skull shape (Fig 3A,B); modern cats have uniformly high bite forces irrespective of body size and apparent, but merely size-related differences in skull shape.

## Discussion

The ability to attain a high gape and administer powerful forces during the killing bite are key factors in predation for all carnivores, but both cannot be optimized simultaneously; in fact, they show a reciprocal relationship, in that, as gape increases, muscle inforce levers decrease, and so do bite forces [4], [6], [29], [30]. The cat family graphically demonstrates this principle, and also demonstrates how divergent evolutionary selective driving forces may be within a relatively narrow taxonomic unit (traditionally called a Family) of animals with an even more restricted feeding ecology and morphological diversity (exclusively meat-eaters; long bodies, powerful limbs, retractile claws; reduced and trenchant post-canine dentition). The results of this study indicate that the cranium and mandible in sabretoothed and feline cats were subjected to fundamentally different selective forces during the course of evolution.

In the modern cat lineage, the primary evolutionary driving force appears to have been uniformly high bite forces, irrespective of body size, enabling these cats to dispatch prey with a powerful killing-bite [3], [31]. Since large predators need large prey for energetic reasons alone [32], [33], this also implied enlargement of the upper canines to facilitate a more deeply penetrating killing bite. Historically, it has been noted that skull morphology appears to differ in large vs. small felid species [24], [26], [34], [35], but evolution of skull shape is tightly coupled with absolute skull size, implying that large pantherines are, in fact, not anatomically different from small species; they are simply larger, and selection for uniformly high bite forces implies elongation and elevation of the posterior part of the skull, and a stronger zygomatic arch to encompass increases in adductor musculature. Brain size in cats scale with slopes far below isometry [36], implying that in small cats, the braincase makes up more of the total skull volume. This relationship, and the accommodation of great adductor musculature to maintain high bite forces to body mass necessitates elongation, elevation, and dorsal straightening of the posterior part of the skull in large species, the latter two due to a large sagittal crest, resulting in a skull shape as observed in large pantherine species and in the puma.

In contrast, sabrecat evolution was strongly directed towards precision killing with very large upper canines, which implies efficient biting at greatly increased gape angles [2], [6], [10], [13], [14]. This led to far greater evolutionary changes in skull and mandible shape than occurred during evolution in the feline cat lineage, probably for functional reasons. Primitive sabrecats had high bite forces, and a skull and mandible morphology which differed from those of most extant felids, but not *Neofelis* sp. [14], [23], [28], [37]. As sabrecats became more specialised, the entire shape of the skull and mandible changed dramatically to facilitate and properly adapt to biting at very high gape angles. This happened at the expense of high bite forces, but in sabrecats, parts of the force driving the canines into the throat of the prey probably came from the upper cervical muscles [2], [11], [12]. The abbreviated, dorsoventrally tall skull, upwards-curving zygomatic arch, and reduced coronoid process were probably adaptations facilitating longer muscle fibres, and, thus higher gape angles [6], [14].

The reasons for such a derived and unusual killing ecology are less clear, but may have had to do with predator competition. During much of the Miocene-Pliocene, sabrecat fossils appear to be more numerous than fossils of feline cats [2], [38]. In modern ecosystems with numerous large, sympatric predators, interspecific harassment, mutual antagonistic behaviours, often resulting in even fatal encounters [39], and kleptoparasitism of kills are very common, and under such circumstances, reduced exposure time is an effective way of reducing the risk of carcass theft [40]–[44]. In many prehistoric ecosystems, predator competition appears to have been more intense than today [42], [45], [46], so rapid killing of prey would have been important, and this could have acted as a selective driving force favouring rapid killing of prey [47], [48]. This could have been the underlying reason for the extreme specializations of derived sabrecats, but eventually proved an ecological *cul de sac*.

Such extreme specialisations indicate predation on large prey exclusively, and make a wider dietary regime, as found in modern large cats [3], [31] unlikely. This is in accord with all available evidence of prey preference among sabrecats, which unanimously indicates predation on large prey [49]–[52]. Additionally, by following this evolutionary route, the sabrecats were apparently unable to exploit the wide size regime of the feline cats, which specialised in powerful precision biting instead, and this technique appears effective against large and small prey alike. To date, no derived sabretoothed cat the size of a lynx, let alone a margay or a sand cat, have been discovered. The sabrecats appear to have been a prime example of strong selective forces for an unusual feeding ecology, resulting in highly derived species, which probably monopolized the large-predator niches so long as the ecosystems and climate zones remained relatively stable. The tradeoffs were powerful bite forces, a narrow dietary and body size regime, collectively probably implying greater risk of extinction if the environmental conditions to which these cats had become specialised changed too much or too rapidly [6], [46], [53].

## Materials and Methods

### Data sample and shape analysis

A sample of extant feline felids of 424 specimens representing 24 different species was studied, and encompassed male and female adult specimens of every species; the sample of extinct, sabretoothed felids consisted of nine species, and 20 crania and 25 mandibles (see Composition of data sample S1). All specimens were digitally photographed in high resolution in the direct lateral perspective with a millimetre scale ruler positioned directly in line with the long axis of the image perspective and the specimen. Specimens had to be near complete and undistorted in lateral view to be of use in digital surface morphometry, and, accordingly, no skull of *Machairodus aphanistus* was included in such analyses, because all known specimens are either highly incomplete or have suffered at least some post-mortem distortion. All included specimens were scaled to a uniform condylobasal skull length or mandibular length, as appropriate, of 100 mm, at an image resolution of 700 dpi. Twenty-two landmarks were digitally scored on each skull and seventeen on each mandible to encompass overall shape (Fig. 1), and Thin Plate Spline (TPS) Relative Warp analyses [54] were conducted on the digitized specimens (see Procedure for digital shape analysis S2). TPS models shape differences as deformations among a set of homologous landmarks, and the TPS function interpolates a surface that is fixed at the landmarks, and is computed so as to minimize overall bending energy, implying minimizing spatially localized information [25], [54]. A non-arbitrary and non-local consensus configuration, defining the point of tangency between shape space and approximating tangent space in the computation of the thin plate splines is computed by the generalized orthogonal least squares Procrustes superimposition procedure [54], [55]. It constitutes an initial consensus shape, and from this, differences in coordinate distances are computed for every specimen. Displacements occur in a two-dimensional (X, Y) plane, but may be visualised as being vertical displacement in the Z-plane. Relative warps summarize the variation in shape among specimens with respect to their partial warp scores, and at total shape space (α = 0), constitute a Principal Components Analysis (PCA) of shape changes based on the covariance matrix of partial warp scores [54], [56]. The relative warps are orthogonal and uncorrelated, and account for virtually all of the variation in the sample [54], [57]. A relative warp analysis is thus similar to a traditional PCA in that relative warp 1 describes most of the variation in the sample, followed by relative warp 2, and so on. The principal difference is that relative warps are coordinate distances of shape variation derived through Procrustes superimposition of specimens [54], [55] and not measured variables, and that the variance captured by the relative warps is therefore related to differences in shape and not linear measurements.

### Bite force estimations and Bite Force Quotients

For this purpose, the included specimens were digitally photographed in high resolution in direct lateral, ventral, and postero-dorsal perspective with a millimetre scale ruler positioned directly in line with the long axis of the image perspective and the specimen. Bite forces were computed from the digital images using Thomason's dry-skull procedure of muscle cross-sectional area estimation and computation of inlever and outlever moments arms [58]. Bite forces are, however, greatly size dependent, and to facilitate comparison among differently-sized specimens, the Bite Force Quotient [5], [59] was computed for every specimen, and average values were used for regression analyses. This requires a body size as well, and this was computed directly from specimens which had been weighed prior to death by allometry comparisons (see Procedure for computation of Bite Force Quotients (BFQ) S3).

### Regression analysis and data transformation

Bivariate Reduced Major Axis (Model II) regression analyses were carried out on the various variables. This procedure was chosen as opposed to traditional Least Squares (Model I) regression, because it does not assign dependence to a given variable (Y), and is further appropriate since uncertainty has to be assumed on both variables, and the included specimens are derived from a larger population [60]. Relative Warp scores and Bite force Quotients were analysed without transformation, condylobasal skull length and mandibular length were logarithmically (log _10_) transformed prior to analysis, and ratios of upper canine height to condylobasal skull length were angular (or arcsine) transformed prior to analysis to restore normality [60].

## Supporting Information

Composition of Data Sample S1 (0.12 MB DOC)
