# Supplementary material for: Evolution of Skull and Mandible Shape in Cats (Carnivora: Felidae)
Source: PLoS One. 2008 Jul 30;3(7):e2807. doi: 10.1371/journal.pone.0002807 (PMC2475670; doi:10.1371/journal.pone.0002807)
Supplement: Composition of Data Sample S1 — (0.12 MB DOC) [file pone.0002807.s001.doc]

**Composition of data sample S1**

A total of 424 specimens representing 24 different species, and spanning the entire size and phylogenetic spectrum within extant felids were included in this study, as shown in the table below. The species *Neofelis diardi* was formerly considered a subspecies of the clouded leopard (*Neofelis nebulosa*), but recent research on genetic [1,2], pelage [3], and craniodental [4] characters have indicated that this form is so different from the clouded leopard that it warrants the rank of separate species, and several craniomandibular and dental characters indicate differences in evolutionary adaptations as well [4]. The included specimens are housed at the American Museum of Natural History, New York; Natural History Museum, London; Zoological Museum, Copenhagen; Museum für Naturkunde, Berlin; Museum National d¨Histoire Naturelle, Paris; National Museum of Natural History (Naturalis), Leiden; Shanghai Science and Technology Museum; Naturmuseum Senckenberg, Frankfurt; and Naturhistoriska riksmuseet, Stockholm.

Nine species of sabertoothed felid were included. The included specimens are: Institut Catalá de Paleontologia in Sabadell, Barcelona: Cast of *Megantereon cultridens* skull (ICP20065); Los Angeles County Museum, Hancock Collection: *Smilodon fatalis* (skulls: LACMHC2001-3, 2001-64, 2001-173, 2001-171, 2001-181, 2001-215; mandibles: LACMH2002-L-552, LACMH2002-L-553, 2002-L-543, 2002-L-545, 2002-L-548, and 2002-L-705); Museum national d’Histoire Naturelle, Paris: *Homotherium crenatidens* (associated skull and mandible: MNHN Per2000a, and MNHN Per2000b, respectively), *Megantereon cultridens* (skull: MNHNPer2001a), *Smilodon fatalis* (skull: MNHN2050-21944), and *Smilodon populator* (mandibles: MNHN1907-13, and MNHN BRD21); Museo Nacional de Ciencias Naturales, Madrid: *Epimachairodus giganteus* (cast; no museum number), *Machairodus aphanistus* (mandibles: MNCN B-2230, and B-4272), and *Paramachairodus ogygia* (skulls: MNCN B-1377, B-5406, and an un-numbered specimen; mandibles: MNCN B-2376, B-3109, B-4708, and B-5198); Natural History Museum, London: *Smilodon populator* (two un-numbered casts of associated skulls and mandibles); Naturhistorisches Museum, Basel: *Megantereon cultridens* (associated skull and mandible: SE311); Texas Memorial Museum, Texas: *Homotherium serum* (associated skull and mandible: TMM933-3582); Transvaal Museum, Pretoria: *Dinofelis barlowi* (associated skull and mandible: BF55-22, and BF55-23, as high-resolution cast UC113720); Zoological Museum, Copenhagen: *Smilodon populator* (skull: CN60; mandibles: CN2, CN4; associated skull and mandible: CN52; un-numbered cast of associated skull and mandible).

Historically, the genus *Machairodus* has not been well-known, and only recently has complete specimens been discovered at the Late Miocene carnivore trap at Batallones near Madrid, Spain [5]. However, whereas one of the skulls is complete enough to permit assessment of bite forces (see below), the Thin Plate Spline analysis requires that no distortion be present in skull shape, and this is not the case even for the most well-preserved skulls of *Machairodus aphanistus*. Only very few skulls are known of *Megantereon cultridens* [6,7], and the hitherto only nearly complete and undistorted skull is from the so-called Dmanisi (Georgia) specimen [8]. A cast of this specimen was used (ICP20065), but the Dmanisi *Megantereon* lacks most of the nasals, so these had to be reconstructed based on the well-preserved skull of SE311 from Basel and MNHNPer2001a from Paris [6]. It also lacks the posterior-most part of the sagittal crest and upper-most part of the occipital crest, and these were reconstructed using the skull MNHNPer2001a, which is well-preserved posteriorly. The retroarticular process on the mandible of *Homotherium crenatidens* was estimated based on the morphology of the mandible in *H. serum*. The cranium of the otherwise well-preserved and undistorted skull of *Dinofelis barlowi* lacks the zygomatic arches, and landmarks 7 and 8, and volume of jaw adductor muscles (see below) were estimated based on other finds with complete zygomatic arches [9,10]. In most specimens of *Smilodon* sp, the mandible lacks the P_3_. However, since landmarks cannot be omitted from some specimens in a Thin Plate Splines analysis, the position of landmark 14 in all specimens of *Smilodon* was estimated based on the few specimens where a P_3_ is present (e.g., MNHN BRD21). When present in *Smilodon* sp., the P_3_ is separated by a distinctly smaller diastema than is present in *Homotherium* sp., whereas there is usually only a small or no diastema among the species of extant felids. This is also the case among less derived sabercats such as *Paramachairodus*, *Machairodus*, and *Dinofelis*.

List of analysed species and numbers of specimens.

Felinae Males Females

*Acinonyx jubatus* 6 8

*Caracal caracal* 4 3

*Catopuma temmincki* 3 3

*Felis chaus* 6 3

*Felis silvestris* 4 7

*Leopardus pardalis* 7 6

*Leopardus tigrina* 7 5

*Leopardus wiedii* 5 5

*Leptailurus serval* 6 5

*Lynx canadensis* 4 6

*Lynx lynx* 6 7

*Oncifelis geoffroyi* 5 5

*Pardofelis marmorata* 4 3

*Prionailurus bengalensis* 5 7

*Prionailurus planiceps* 4 4

*Prionailurus viverrinus* 6 4

*Puma concolor* 12 8

Pantherinae

*Neofelis diardi* 13 8

*Neofelis nebulosa* 7 5

*Panthera leo*  28 21

*Panthera onca* 22 18

*Panthera pardus* 28 16

*Panthera tigris* 25 25

*Panthera uncia* 10 15

Crania Mandibles

Machairodontinae

*Dinofelis barlowi* 1 1

*Epimachairodus giganteus* 1 1

*Homotherium crenatidens* 1 1

*Homotherium serum* 1 1

*Machairodus aphanistus* 0 2

*Megantereon cultridens* 1 1

*Paramachairodus ogygia* 3 4

*Smilodon fatalis* 7 6

*Smilodon populator* 5 8

**Procedure for digital shape analysis S2**

The shape of the skull and mandible was analysed in the included specimens by means of the 2D or surface morphometry, Thin Plate Spline procedure [11]. The thin-plate spline decomposed by its partial warps analyses shape deformation of a structure fixed at a number of homologous landmarks compared to a predefined reference shape configuration [11,12]. The landmarks were digitized using the program tpsDig [13] onto a high-resolution image (700 dpi) of every specimen that had already been re-scaled to a standard size of 100 mm in condylobasal length (skull) or symphysis-mandible condyle length (mandible), as appropriate. Shape comparisons by means of Thin Plate Spline analysis were conducted in tpsRelw [14].

Initially, a non-arbitrary and non-local reference configuration is computed, defining the point of tangency between shape space and approximating tangent space in the computation of the thin-plate splines, by means of the generalized orthogonal least squares Procrustes superimposition procedure [10,15-17]. It is a mean shape configuration of the included specimens, and, accordingly, it has no morphological, ontogenetic or phylogenetic significance, and simply serves as a standard for comparison among the specimens under analysis.

The resulting bending energy when going from the shape configuration of the reference structure to any given specimen under analysis is a function of the distance between landmarks of the reference configuration and the particular specimen in question, and it increases progressively with increased localization, i.e. changes affecting only a limited area of the structure [11,12]. The thin-plate spline function

n

f_(x,y)_ = *a_x_x* + *a_y_y* + *a_o_* + Σ*w_i_U*(│*(x,y)* - *P_i_*│)

i=1

interpolates a surface which is fixed at the landmarks, and is computed so as to minimize the overall bending energy function

⌠⌠ (*f^2^_xx_* + *f^2^_yy_* + 2*f^2^_xy_*)*d_x_d_y_*

R^2^

where bending energy is defined as the integral over R^2^ of the squares of the second derivatives, f_x_ and f_y_ are the separate thin-plate spline functions, w_i_ are coordinates, and U is r^2^log(r^2^), which is the so-called fundamental solution to the biharmonic equation △*^2^U* = 0.

This has important implications for parsimonious interpretation of the analysis results, because although any morphological change may be interpreted as a singular event, such interpretation is, by default, un-parsimonious. In thin plate splines analysis, changes are interpreted as localised only if the surrounding landmarks indicate it is, in fact, confined to the area in question. Spatially correlated changes are interpreted as a single feature. Thus, minimising bending energy equals minimising the amount of spatially localised information, and, accordingly, implies a more conservative and parsimonious interpretation of morphological change, in that characters are interpreted as evolving independently only when the data require it [11,16,17].

Displacements are of two basic kinds, affine and non-affine. Affine changes affect the entire structure in tilting, rotation or elevation; such changes have a bending energy of zero, since they involve no displacement of landmarks relative to other landmarks. Non-affine changes are localised changes in one region relative to others; they may be decomposed by eigenanalyses of a bending energy matrix into a series of non-affine components of a progressively more localised nature. The resulting principal warps are geometric properties describing morphological differences between each specimen and the reference configuration, and are computed from the bending energy matrix, projecting each specimen onto them to yield the partial warps. These are a set of variables where the reference shape is decomposed into affine and non-affine components of the final form, and, thus, describe both uniform and non-uniform shape changes, which are given as X-Y coordinates.

The relative warps are a summary of the variation among the included specimens with respect to their partial warp scores, and at an α=0 constitute a Principal Components Analysis (PCA) of shape changes based on the covariance matrix of partial warp scores [18]. The relative warps are orthogonal and uncorrelated, and account for virtually all of the variation in the sample [11,17]. Accordingly, the relative warp analysis is similar to a traditional PCA in that relative warp 1 describes most of the variation in the sample, followed by relative warp 2, and so on.

Below are given the variances for the landmarks used in the analysis, along with post-hoc ANOVA comparisons with Bonferroni adjustment for relative warps 1 and 2.

Variances at each landmark for aligned specimens in the analysis of skull shape:

Ni S²x S²y S²

1 0.1073 0.0160 0.1233

2 0.0964 0.0001 0.0965

3 0.0770 0.0042 0.0812

4 0.0756 0.0001 0.0757

5 0.0424 0.0010 0.0434

6 0.0357 0.0002 0.0359

7 0.0148 0.0015 0.0163

8 0.0003 0.0024 0.0027

9 0.0026 0.0024 0.0049

10 0.0168 0.0009 0.0177

11 0.0037 0.0101 0.0139

12 0.0196 0.0078 0.0274

13 0.0345 0.0068 0.0413

14 0.0482 0.0067 0.0550

15 0.0737 0.0047 0.0783

16 0.0900 0.0056 0.0956

17 0.0586 0.0037 0.0623

18 0.0121 0.0182 0.0303

19 0.0007 0.0318 0.0325

20 0.0138 0.0322 0.0460

21 0.0027 0.0042 0.0070

22 0.0230 0.0001 0.0231

Variances at each landmark for aligned specimens in the analysis of mandible shape:

Ni S²x S²y S²

1 0.1211 0.0005 0.1216

2 0.0821 0.0016 0.0837

3 0.0040 0.0028 0.0068

4 0.1021 0.0474 0.1495

5 0.1088 0.0103 0.1192

6 0.0583 0.0120 0.0703

7 0.0003 0.0013 0.0016

8 0.0008 0.0020 0.0027

9 0.0006 0.0109 0.0116

10 0.0059 0.0012 0.0071

11 0.0069 0.0106 0.0176

12 0.0270 0.0012 0.0282

13 0.0292 0.0086 0.0379

14 0.0502 0.0021 0.0522

15 0.0535 0.0073 0.0607

16 0.0896 0.0059 0.0955

17 0.1401 0.0052 0.1453

Singular values, percent of variance explained, and cumulative percent variance explained for the first 20 relative warps in the analysis of skull shape:

No. SV % Cum %

1 1.03976 40.12% 40.12%

2 0.73336 19.96% 60.08%

3 0.48477 8.72% 68.80%

4 0.36085 4.83% 73.64%

5 0.31379 3.65% 77.29%

6 0.29550 3.24% 80.53%

7 0.26255 2.56% 83.09%

8 0.25573 2.43% 85.52%

9 0.22076 1.81% 87.33%

10 0.19047 1.35% 88.67%

11 0.17861 1.18% 89.86%

12 0.16660 1.03% 90.89%

13 0.14888 0.82% 91.71%

14 0.14419 0.77% 92.48%

15 0.13650 0.69% 93.17%

16 0.13508 0.68% 93.85%

17 0.12973 0.62% 94.47%

18 0.12348 0.57% 95.04%

19 0.11992 0.53% 95.57%

20 0.11816 0.52% 96.09%

Singular values, percent of variance explained, and cumulative percent variance explained for the first 20 relative warps in the analysis of mandible shape:

No. SV % Cum %

1 0.98631 50.69% 50.69%

2 0.59020 18.15% 68.84%

3 0.38003 7.53% 76.36%

4 0.28832 4.33% 80.70%

5 0.26012 3.53% 84.22%

6 0.24267 3.07% 87.29%

7 0.21336 2.37% 89.66%

8 0.20214 2.13% 91.79%

9 0.15734 1.29% 93.08%

10 0.15392 1.23% 94.31%

11 0.13323 0.92% 95.24%

12 0.11667 0.71% 95.95%

13 0.11414 0.68% 96.63%

14 0.09829 0.50% 97.13%

15 0.09519 0.47% 97.60%

16 0.08741 0.40% 98.00%

17 0.08359 0.36% 98.37%

18 0.07901 0.33% 98.69%

19 0.07479 0.29% 98.98%

20 0.06673 0.23% 99.21%

*Post hoc* Tukey HSD comparison of relative warp scores 1 and 2 for analysis on skull shape.

**Sabertoothed cats**:

Symbols:

1: *Dinofelis barlowi*

2: *Epimachairodus giganteus*

3: *Homotherium crenatidens*

4: *Homotherium serum*

5: *Megantereon cultridens*

6: *Paramachairodus ogygia*

7: *Smilodon fatalis*

8: *Smilodon populator*

Relative warp 1

1 2 3 4 5 6 7 8

1 1.000

2 0.002 1.000

3 0.001 0.999 1.000

4 0.013 0.899 0.652 1.000

5 0.056 0.433 0.221 0.980 1.000

6 0.995 0.000 0.000 0.001 0.005 1.000

7 0.000 1.000 0.998 0.670 0.150 0.000 1.000

8 0.000 1.000 0.999 0.651 0.149 0.000 1.000 1.000

Relative warp 2

1 2 3 4 5 6 7 8

1 1.000

2 0.993 1.000

3 0.943 1.000 1.000

4 0.807 0.994 1.000 1.000

5 0.965 1.000 1.000 1.000 1.000

6 1.000 0.989 0.897 0.682 0.937 1.000

7 0.021 0.103 0.215 0.411 0.175 0.001 1.000

8 0.025 0.119 0.241 0.446 0.198 0.001 1.000 1.000

**Extant cats by genus**:

Symbols:

1: *Acinonyx*

2: *Caracal*

3: *Catopuma*

4: *Felis*

5: *Leopardus*

6: *Leptailurus*

7: *Lynx*

8: *Oncifelis*

9: *Pardofelis*

10: *Prionailurus*

11: *Puma*

12: *Neofelis*

13: *Panthera*

Relative warp 1

1 2 3 4 5 6 7 8 9 10 11 12 13

1 1.000

2 0.027 1.000

3 0.263 1.000 1.000

4 0.000 1.000 1.000 1.000

5 0.000 1.000 1.000 0.998 1.000

6 0.000 1.000 1.000 0.999 1.000 1.000

7 0.000 1.000 1.000 1.000 0.250 0.629 1.000

8 0.000 1.000 1.000 1.000 1.000 1.000 0.883 1.000

9 0.335 1.000 1.000 1.000 1.000 1.000 1.000 1.000 1.000

10 0.000 1.000 1.000 0.999 1.000 1.000 0.331 1.000 1.000 1.000

11 0.003 0.973 0.997 0.885 0.000 0.050 0.998 0.340 0.999 0.002 1.000

12 0.000 0.980 0.998 0.901 0.000 0.040 0.999 0.349 0.999 0.001 1.000 1.000

13 0.985 0.000 0.051 0.000 0.000 0.000 0.000 0.000 0.075 0.000 0.000 0.000 1.000

Relative warp 2

1 2 3 4 5 6 7 8 9 10 11 12 13

1 1.000

2 0.664 1.000

3 0.139 0.993 1.000

4 0.000 0.994 1.000 1.000

5 0.000 0.017 0.994 0.034 1.000

6 0.001 1.000 0.999 1.000 0.000 1.000

7 0.001 1.000 0.997 0.991 0.000 1.000 1.000

8 0.000 0.120 0.999 0.435 1.000 0.029 0.006 1.000

9 0.019 0.868 1.000 0.998 1.000 0.934 0.868 1.000 1.000

10 0.000 0.178 1.000 0.552 0.867 0.007 0.000 1.000 1.000 1.000

11 0.001 1.000 0.992 0.948 0.000 1.000 1.000 0.001 0.800 0.000 1.000

12 0.000 0.893 1.000 1.000 0.000 0.764 0.276 0.325 1.000 0.117 0.019 1.000

13 0.000 1.000 0.987 0.857 0.000 1.000 1.000 0.000 0.750 0.000 1.000 0.000 1.000

**Extant cats; *Panthera* genus only**:

Symbols:

1: *Panthera leo*

2: *Panthera onca*

3: *Panthera pardus*

4: *Panthera tigris*

5: *Panthera uncia*

Relative warp 1

1 2 3 4 5

1 1.000

2 0.000 1.000

3 0.000 0.075 1.000

4 0.000 0.000 0.000 1.000

5 0.000 0.006 0.000 0.000 1.000

Relative warp 2

1 2 3 4 5

1 1.000

2 0.008 1.000

3 0.010 1.000 1.000

4 0.000 0.000 0.000 1.000

5 0.998 0.094 0.118 0.000 1.000

*Post hoc* Tukey HSD comparison of relative warp scores 1 and 2 for analysis on mandible shape.

**Sabertoothed cats**:

Symbols:

1: *Dinofelis barlowi*

2: *Epimachairodus giganteus*

3: *Homotherium crenatidens*

4: *Homotherium serum*

5: *Machairodus aphanistus*

6: *Megantereon cultridens*

7: *Paramachairodus ogygia*

8: *Smilodon fatalis*

9: *Smilodon populator*

Relative warp 1

1 2 3 4 5 6 7 8 9

1 1.000

2 0.468 1.000

3 0.063 0.927 1.000

4 0.346 1.000 0.976 1.000

5 1.000 0.556 0.058 0.405 1.000

6 0.088 0.969 1.000 0.993 0.086 1.000

7 1.000 0.112 0.005 0.066 0.951 0.009 1.000

8 0.002 0.382 0.999 0.552 0.000 0.989 0.000 1.000

9 0.008 0.773 1.000 0.909 0.002 1.000 0.000 0.868 1.000

Relative warp 2

1 2 3 4 5 6 7 8 9

1 1.000

2 0.667 1.000

3 1.000 0.818 1.000

4 0.998 0.281 0.981 1.000

5 0.991 0.939 1.000 0.731 1.000

6 1.000 0.890 1.000 0.953 1.000 1.000

7 0.662 1.000 0.848 0.207 0.962 0.927 1.000

8 0.521 1.000 0.734 0.132 0.875 0.846 1.000 1.000

9 0.998 0.729 1.000 0.751 1.000 1.000 0.501 0.210 1.000

**Extant cats by genus**:

Symbols:

1: *Acinonyx*

2: *Caracal*

3: *Catopuma*

4: *Felis*

5: *Leopardus*

6: *Leptailurus*

7: *Lynx*

8: *Oncifelis*

9: *Pardofelis*

10: *Prionailurus*

11: *Puma*

12: *Neofelis*

13: *Panthera*

Relative warp 1

1 2 3 4 5 6 7 8 9 10 11 12 13

1 1.000

2 0.041 1.000

3 0.031 1.000 1.000

4 0.000 0.291 0.997 1.000

5 0.000 1.000 1.000 0.027 1.000

6 0.000 1.000 1.000 0.040 1.000 1.000

7 0.000 0.377 1.000 1.000 0.010 0.032 1.000

8 0.000 0.976 1.000 0.959 0.982 0.953 0.990 1.000

9 0.017 0.999 1.000 0.999 1.000 1.000 1.000 1.000 1.000

10 0.000 0.516 1.000 0.999 0.006 0.041 1.000 0.999 1.000 1.000

11 0.000 0.999 1.000 0.121 1.000 0.999 0.090 0.999 1.000 0.099 1.000

12 0.000 0.000 0.010 0.000 0.000 0.000 0.000 0.000 0.019 0.000 0.000 1.000

13 0.000 0.009 0.893 0.995 0.000 0.000 0.567 0.125 0.950 0.008 0.000 0.000 1.000

Relative warp 2

1 2 3 4 5 6 7 8 9 10 11 12 13

1 1.000

2 1.000 1.000

3 0.991 0.999 1.000

4 0.680 0.990 1.000 1.000

5 0.000 0.149 0.999 0.555 1.000

6 0.031 0.743 1.000 0.999 0.970 1.000

7 0.931 1.000 1.000 1.000 0.007 0.792 1.000

8 0.044 0.594 1.000 0.988 1.000 1.000 0.667 1.000

9 1.000 1.000 1.000 1.000 0.978 1.000 1.000 0.998 1.000

10 0.000 0.059 0.993 0.235 1.000 0.715 0.001 0.999 0.917 1.000

11 0.668 0.987 0.663 0.004 0.000 0.000 0.005 0.000 0.909 0.000 1.000

12 1.000 1.000 0.923 0.090 0.000 0.000 0.160 0.001 0.994 0.000 0.872 1.000

13 0.382 0.979 0.618 0.001 0.000 0.000 0.000 0.000 0.889 0.000 1.000 0.312 1.000

**Extant cats; *Panthera* genus only**:

Symbols:

1: *Panthera leo*

2: *Panthera onca*

3: *Panthera pardus*

4: *Panthera tigris*

5: *Panthera uncia*

Relative warp 1

1 2 3 4 5

1 1.000

2 0.268 1.000

3 0.000 0.242 1.000

4 0.999 0.361 0.000 1.000

5 0.978 0.843 0.036 0.993 1.000

Relative warp 2

1 2 3 4 5

1 1.000

2 0.874 1.000

3 0.005 0.002 1.000

4 0.000 0.000 0.000 1.000

5 0.000 0.000 0.011 0.000 1.000

**Procedure for computation of Bite Force Quotients (BFQ) S3**

Body masses were not taken as simple literature values, as is traditionally done; this implies no direct correlation of any skeletal specimen size with assigned body mass. In all included species of extant cats, at least some specimens had been weighed prior to, or immediately after death. Body masses were assigned to all unweighted specimens by allometric extrapolation of condylobasal skull length; where possible, the computation was made gender-specific. For instance, out of 40 jaguars, 6 had been weighed prior to death (2 females; 4 males). Condylobasal length in the two female jaguars (CN5659: CBL: 219.8 mm, mass: 47.3 kg; and CN5707: CBL: 222.4, mass: 51.5 kg) were used to assign body masses to the other 16 females, for instance NRM583362, with a CBL of 210.8 mm. This specimens’ body mass was computed as (210.8/219.8)^3^*47.3 kg, and (210.8/222.4)^3^*51.5 kg, resulting in 41.7 kg, and 43.9 kg, respectively. The final value for NRM583362 was then (41.7 + 43.9 kg)/2 = 42.8 kg; and so on for all females. For males, the four male specimens which had been weighed were used in the same way. In some small cats, e.g., flat-headed cat (2 female), serval (3 males), margay (1 female), weighed specimens were of the same sex, and so had to be used on all included specimens.

Bite forces were computed using Thomason’s [19] dry skull model of assessment of muscle cross-sectional areas, and inlever and outlever moment arms, and were computed at the canine, using the linear distance from the centre of the canine at the alveolar margin–centre of glenoid fossa, as outlever; the isometric value of 350 Kpa from [20] was used instead of the usual 300 Kpa, which in this model leads to underestimation of bite forces. Bite forces thus computed are returned in Newtons, and are, naturally, going to be extremely size-dependent and thus not readily comparable across species with different body sizes. Wroe et al. [21] presented a novel approach for comparison of bite forces among species of different body sizes, the Bite Force Quotient, or BFQ. This procedure was used to assign a BFQ value to every specimen in the study, and averages (ΣBFQ/n) within each species were used in subsequent regression analyses.

Bite forces and BFQ’s for the included taxa of sabertoothed cats were taken from [22], except for *Dinofelis barlowi*, which was computed for the current study based on the regression equations in the former study. Reconstruction of muscle cross-sectional areas was tentative in *Dinofelis barlowi*, owing to the incompleteness of the zygomatic arches in the included specimen. However, muscle areas were assessed by using other specimens, which had complete zygomatic arches in at least one side of the skull [9,10]. In *Paramachairodus ogygia*, bite forces were not computed in B-5406, owing to lateral compression of the zygomatic arches, making masseter cross-sectional area and inlever moment arms too uncertain.

Average condylobasal skull length (CBL) and upper canine heights (CH; both in mm), and Bite Force Quotients (BFQ) at the canine for the included species.

CBL CH BFQ

Extant cats

148.9 22.8 105.9 *Acinonyx jubatus*

106.7 16.2 115.2 *Caracal caracal*

113.1 17.1 92.1 *Catopuma temmincki*

96.8 14.0 94.2 *Felis chaus*

80.6 11.1 127.4 *Felis silvestris*

126.9 18.4 94.6 *Leopardus pardalis*

84.1 10.3 94.5 *Leopardus tigrina*

87.4 11.3 91.9 *Leopardus wiedii*

109.7 14.6 93.9 *Leptailurus serval*

129.4 22.6 93.1 *Lynx lynx*

116.4 18.7 102.1 *Lynx canadensis*

146.1 35.1 121.5 *Neofelis diardi*

147.9 30.9 122.0 *Neofelis nebulosa*

94.2 13.6 101.5 *Oncifelis geoffroyi*

302.9 49.0 89.2 *Panthera leo*

224.5 43.5 117.0 *Panthera onca*

191.9 37.0 98.5 *Panthera pardus*

273.7 56.1 99.3 *Panthera tigris*

166.3 30.1 96.7 *Panthera uncia*

89.8 13.9 104.3 *Pardofelis marmorata*

82.5 11.1 99.2 *Prionailurus bengalensis*

96.2 13.8 92.7 *Prionailurus planiceps*

118.1 17.2 103.1 *Prionailurus viverrinus*

178.7 29.2 108.0 *Puma concolor*

Sabertoothed cats

239.4 51.5 67.5 *Dinofelis barlowi*

316.6 99.8 61.2 *Epimachairodus giganteus*

300.4 95.1 31.6 *Homotherium crenatidens*

297.8 74.2 34.8 *Homotherium serum*

257.4 93.5 30.6 *Megantereon cultridens*

170.5 42.0 115.1 *Paramachairodus ogygia*

289.3 141.5 51.4 *Smilodon fatalis*

334.4 176.5 45.7 *Smilodon populator*

**References**

1. Buckley-Beason VA, Johnson WE, Nash WG, Stanyon R, Menninger JC et al (2006) Molecular evidence for species-level distinctions in clouded leopards. Current Biol. 16: 2371-2376.

2. Wilting A, Buckley-Beason VA, Feldhaar H, Gadau J, O´Brien SJ et al (2007) Clouded leopard phylogeny revisited: support for species recognition and population division between Borneo and Sumatra. Front Zool 4: 1-10.

3. Kitchener AC, Beaumont MA, Richardson D (2006) Geographical variation in the clouded leopard, *Neofelis nebulosa*, reveals two species. Current Biol. 16: 2377-2383.

4. Christiansen, P. (2008) Species distinction and evolutionary differences in the clouded leopard (*Neofelis nebulosa*) and Diard’s leopard (*Neofelis diardi*). J Mammal.

5. Antón M, Salesa MJ, Morales J, Turner A (2004) First known complete skulls of the scimitar-toothed cat *Machairodus aphanistus* (Felidae, Cranivora) from the Spanish Late Miocene site of Batallones-1. J Vert Paleontol 24: 957-969.

6. Christiansen P, Adolfssen JS (2007) Osteology and ecology of *Megantereon cultridens* SE311 (Mammalia; Felidae; Machairodontinae), a sabrecat from the Late Pliocene-Early Pleistocene of Senéze, France. Zool J Linn Soc 151: 833-884.

7. Palmquist P, Torregrosa V, Pérez-Claros JA, Martínez-Navarro B, Turner A (2007) A re-evaluation of the diversity of *Megantereon* (Mammalia, Carnivora, Machairodontinae) and the problem of species identification in extinct carnivores. J Vert Paleontol 27: 160-175.

8. Vekua A. (1995) Die Wirbeltierfauna des Villafranchium von Dmanisi und ihre Biostratigraphische Bedeutung. Jahrb Römisch-Germanischen Zentralmus Mainz 42: 77-180.

9. Cooke HBS (1991) *Dinofelis barlowi* (Mammalia, Carnivora, Felidae) cranial material from Bolt’s Farm, collected by the University of California African Expedition. Palaeontol Afr 28: 9-21.

10. Werdelin L, Lewis ME (2001) A revision of the genus *Dinofelis* (Mammalia, Felidae). Zool J Linn Soc 132: 147-258.

11. Bookstein FL (1991) Morphometric tools for landmark analysis: Geometry and biology. New York: Cambridge University Press

12. Bookstein FL (1989) Principal warps: thin-plate splines and the decomposition of deformation. IEEE Trans Pattern Anal Mach Intell 11: 567-585.

13. Rohlf FJ (2004) tpsDig, digitize landmarks and outlines, version 2.0. New York: Department of Ecology and Evolution, State University of New York at Stony Brook. [computer program and documentation].

14. Rohlf FJ (2004) tpsSpline, Thin-Plate Spline, version 1.20. New York: Department of Ecology and Evolution, State University of New York at Stony Brook. [computer program and documentation].

15. Chapman RE (1990) Conventional Procrustes approaches. In: Rohlf FJ, Bookstein FL, editors. Proceedings of the Michigan Morphometrics Workshop. Ann Arbor, Michigan: University of Michigan Museum of Zoology. Pp. 252-267.

16. Rohlf FJ, Slice DE (1990) Extensions of the Procrustes method for the optimal superimposition of landmarks. Syst Biol 39: 40-59.

17. Bookstein FL (1996) Combining the tools of geometric morphometrics. In: Marcus LF, Corti M, Loy A, Naylor GJP, Slice DE, editors. Proceedings of the 1993 NATO Advanced Studies Institute on Morphometrics, IL Ciocco, Italy. New York: Plenum Publishing Corporation. Pp. 131-151.

18. Rohlf FJ (1993) Relative warp analysis and an example of its application to mosquito wings. In: Marcus LF, Bello E, Garcia-Valdecasas A, editors. Contributions to morphometrics, vol. 8. Madrid: Museo Nacional de Ciencias Naturales (CSIC). Pp. 131-159.

19. Thomason JJ (1991) Cranial strength in relation to estimated biting forces in some mammals. Can J Zool 69: 2326-2333.

20. Christiansen P, Adolfssen JS (2005) Bite forces, canine strengths and skull allometry in extant carnivores (Mammalia, Carnivora). J Zool Lond 266: 1-19.

21. Wroe S, McHenry C, Thomason JJ (2005) Bite club: Comparative biteforce in big biting mammals and the prediction of predatory behaviour in fossil taxa. Proc Royal Soc Lond 272: 619-625.

22. Christiansen P (2007) Comparative biteforces and canine bending strengths in feline and sabretooth felids: implications for predatory ecology. Zool J Linn Soc 151: 423-437.
